# Dedicated Upper Extremity Trauma List for Closed Distal Radius Fractures Improves Surgical Efficiency and Reduces Costs

**DOI:** 10.3390/jcm15218661

**Published:** 2026-11-06

**Authors:** Klaas Victor, Bryan J. M. van de Wall, Beat Galliker, Reto Babst, Jörg Bucher, Björn-Christian Link, Frank J. P. Beeres

**Affiliations:** 1 Lucerne Cantonal Hospital, 6004 Lucerne, Switzerlandreto.babst@luks.ch (R.B.);; 2 Faculty of Health Sciences and Medicine, University of Lucerne, 6005 Lucerne, Switzerland

**Keywords:** dedicated trauma list, upper extremity trauma, distal radius fracture, open reduction and internal fixation, operative time, cost, efficiency

## Abstract

**Introduction**: The operative treatment of closed distal radius fractures has traditionally been managed through trauma waiting lists, resulting in the planning of add-on cases after urgent trauma or elective surgery. We investigated the feasibility of a once-weekly dedicated operating list for the treatment of semi-elective upper extremity fractures and assessed the effects on costs and operative time. **Methods**: A retrospective analysis was performed comparing consecutive periods before and after the introduction of a dedicated upper extremity trauma list at a Level I trauma center. The preimplementation period extended from January 2024 to August 2024, and the postimplementation period from September 2024 to March 2025. The dedicated operating list was run once-weekly and used to schedule the surgical treatment of isolated closed distal radius fractures and clavicle fractures. Closed distal radius fractures that required open reduction and internal fixation (ORIF) via volar plate osteosynthesis accounted for the highest caseload and were reviewed for the purpose of this study. Exclusion criteria were age <18 years, fractures managed through a dorsal approach, associated ligamentous injury, inability to ambulate, and referral >14 days after the date of injury. The operating list was established to facilitate a standardized workflow managed by a dedicated surgical team responsible for treating these fractures on a weekly basis. The consecutive patient cohort from the postimplementation period was compared with a consecutive cohort from the preimplementation period. The following data were collected from the health records: surgical and operating room time per case, procedure- and admission-related costs, time to treatment, hospital stay, and complications and reoperations at the 90-day mark. **Results**: The nondedicated cohort comprised a total of 143 patients that were treated during the preimplementation period. The dedicated cohort comprised 104 consecutive patients treated during the postimplementation period. After introduction of the dedicated upper extremity trauma list, the median surgical time per case was reduced by 51%, from 79 min (range 27–193) to 39 min (range 23–93) (*p* < 0.01). The quartile deviation of surgical time in the nondedicated and dedicated cohorts decreased from 23 to 7 min, respectively (*p* < 0.001). The median total cost per case decreased by 29% (*p* < 0.01). The median time to treatment increased from 2 days to 5 days after implementation of the dedicated list (*p* <0.001). The complication rates at 90 days in the nondedicated and dedicated cohorts were 9.4% and 7.7%, respectively (*p* = 0.82). The reoperation rates at 90 days were 4.3% and 1.9%, respectively (*p* = 0.47). **Conclusion**: A dedicated upper extremity trauma list, run once-weekly by a specialized team, significantly improves time and cost efficiency in volar locked plating of isolated, closed distal radius fractures. **Summary**: This retrospective study evaluated the feasibility and outcomes of introducing a dedicated weekly upper extremity trauma list at a Level I trauma center. A cohort of 143 consecutive patients who underwent volar locked plating of the distal radius in the preimplementation period was compared to a cohort of 104 patients in the postimplementation period. The median surgical time per case, operating room time and overall case time were significantly reduced by 51%, 49% and 38%, respectively, after implementation of the dedicated operating list. The median total cost per case was reduced by 29%. No statistically significant differences in complications and reoperations were observed between the dedicated and nondedicated cohorts at 90-day follow-up; however, noninferiority could not be established.

## 1. Introduction

The practice of orthopedic trauma surgery is confronted with an increase in fractures, due to prolonged life expectancy and an increase in recreational activities. The majority of these fractures do not require urgent hospital admission and can be successfully treated through planned inpatient surgery. Given the less urgent nature of these semi-elective surgical fractures, a term introduced by Prof. Lindsey in 2006, they are designated low-priority when it comes to planning and often booked as add-on cases in a non-specialized setting [1]. As the quality of surgical treatment determines function in the long term, as well as activity level and social participation [2], the growing pool of patients with semi-elective surgical fractures deserves treatment by a highly specialized team [3]. However, the opportunities to provide subspecialized trauma care are often neglected. This is at odds with the current trend of subspecialization that is seen in elective orthopedic surgery, particularly in joint replacement surgery [4,5].

The strength of clustering patients with a common uncomplicated pathology and planning their similar procedures continuously on the same day allows a dedicated surgical team to fully commit to these procedures. This decreases preparation time, procedure time and turnover time [5,6,7], which in turn improves outcomes and lowers the risk of complications and reoperations [4].

In trauma surgery, the principle of clustering similar cases on dedicated operating days is not common practice. However, it may be perfectly organizable for semi-elective surgical fractures and beneficial in terms of efficiency and clinical outcomes, in particular, for closed upper extremity fractures without neurovascular compromise [8].

At our institution, we introduced the dedicated upper extremity trauma day, a once-weekly 8-hour operating list that was solely used to plan surgical treatment of predefined fracture types of the upper extremity. We aimed to assess the effects on the most commonly performed upper extremity procedure in the trauma department at our institution, volar locked plating of the distal radius. We hypothesized that the implementation of a dedicated upper extremity trauma day would be practically feasible and that it would be time- and cost-efficient.

## 2. Methods

### 2.1. Study Design

This single-institution retrospective pre- and postimplementation study was performed at a Level 1 trauma center in Switzerland. The study retrospectively assessed outcomes (including costs, surgical and periprocedural duration, time to treatment, hospital stay, complications and reoperations) before and after the implementation of a dedicated upper extremity trauma day, a once-weekly 8-hour operating list on a designated hospital campus that was exclusively used to plan semi-elective surgical fractures of the upper extremity, including but not limited to volar locked plating of closed distal radius fractures and open reduction and internal fixation of the clavicle fractures. Volar locked plating of the distal radius was selected as the index procedure to be assessed in a pre- and postimplementation cohort of consecutive patients, as it is the upper extremity trauma procedure with the highest caseload at this institution. Other procedures, including open reduction and internal fixation of the clavicle, were not included in the assessed cohorts. The preimplementation period ran from January 2024 to August 2024, and the postimplementation period from September 2024 to March 2025.

Patients from the pre- and postimplementation periods were eligible for retrospective analysis if the following criteria were met: age ≥ 18 years; ASA I-III; closed fracture of the distal radius with or without involvement of the ulnar styloid; treated with volar locked plating through a modified Henry approach, with or without ulnar styloid fixation; and documented general consent for the retrospective processing of health record data. Exclusion criteria were: open fracture; multiple injuries; posttraumatic neurological or vascular deficits; previous fracture at the operated radius; and inability to ambulate autonomously prior to injury. Distal radius fractures with associated ligamentous injuries requiring operative treatment or treated through a dorsal approach were excluded.

The study was conducted in accordance with the Declaration of Helsinki and approved by the Northwest and Central Switzerland Ethics Committee (EKNZ) (project ID: 2026-02204).

### 2.2. Nondedicated Cohort in the Preimplementation Period

**Figure 1:**
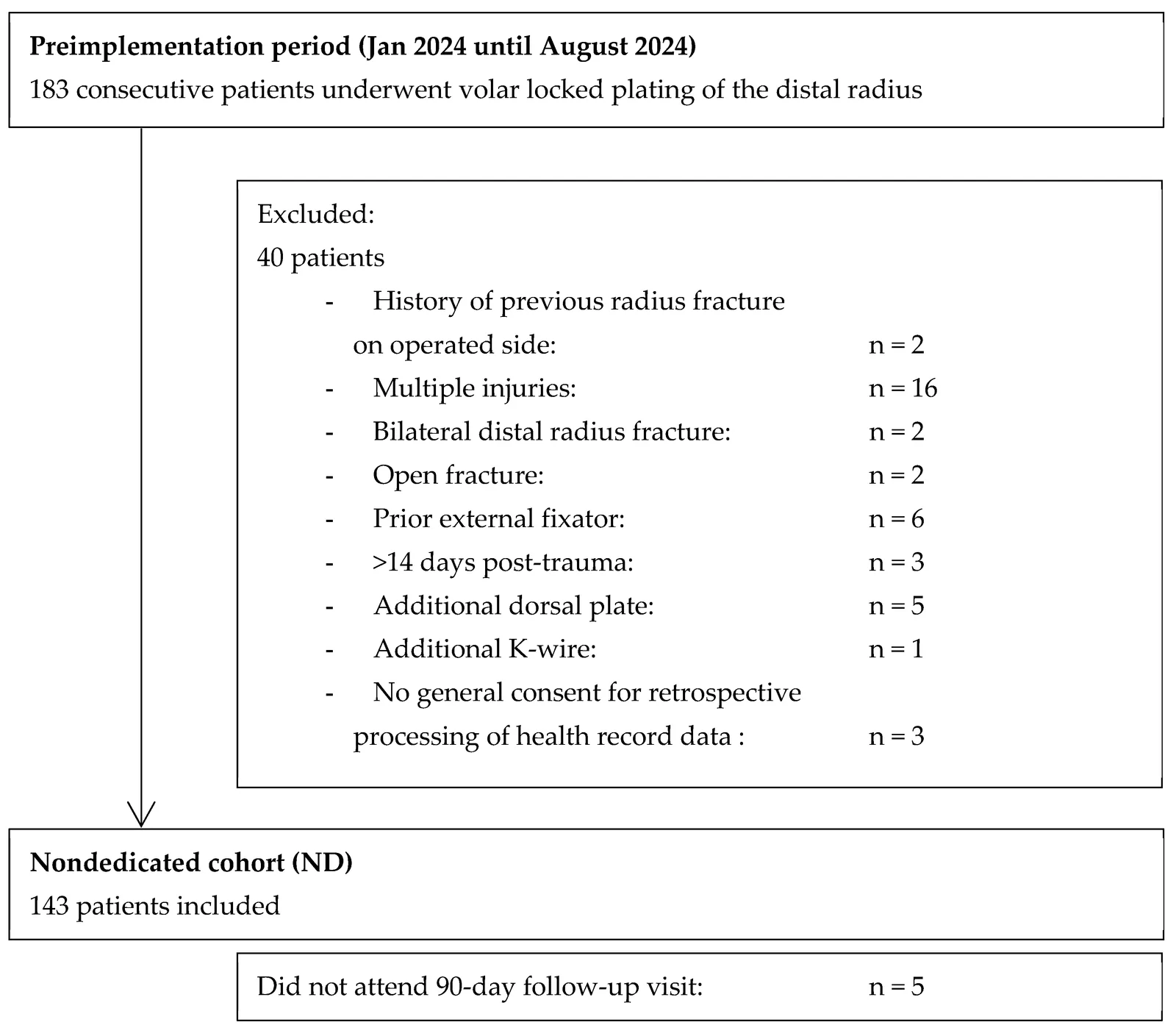
Inclusion pathway during the preimplementation period.

During the preimplementation period, patients requiring volar locked plating of the distal radius were added to a trauma waiting list, along with other trauma patients requiring surgery. These cases were then ranked in order of surgical priority and planned accordingly in an unbooked trauma operating room. This resulted in heterogeneous operating lists, including life- and limb-threatening trauma, combined with lower and upper extremity trauma cases with a variable grade of emergency (Table 1). If highly urgent cases came in during the day, semi-elective surgical fractures, including isolated distal radius fractures requiring volar locked plating, were postponed to another operating room time slot. In the preimplementation period, a group of 10 surgeons performed the procedures using 5 different types of volar locking plate systems. All eligible consecutive patients from the preimplementation period were retrospectively assigned to the nondedicated cohort for comparative analysis (Figure 1).

### 2.3. Dedicated Cohort in the Postimplementation Period

**Figure 2:**
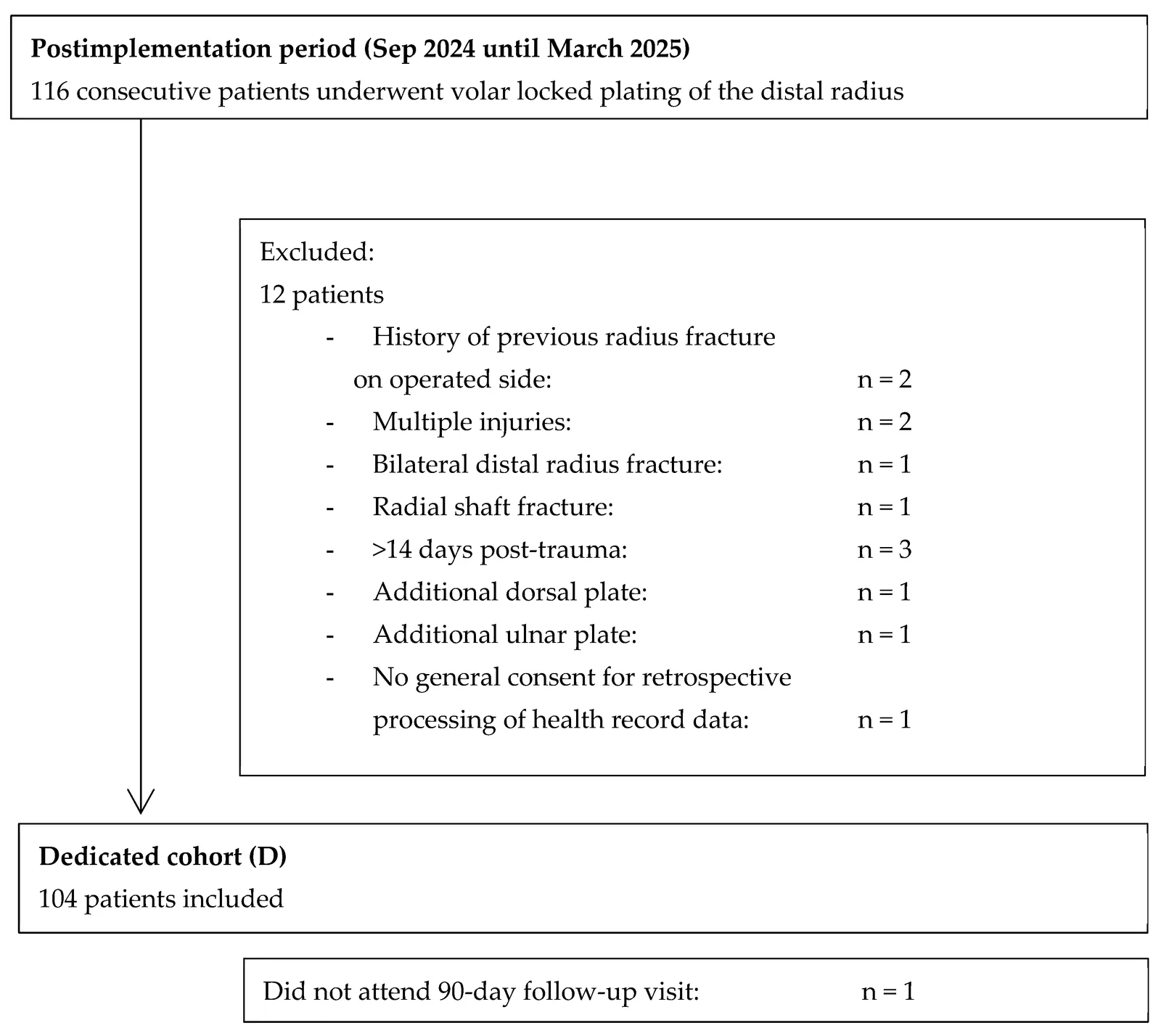
Inclusion pathway during the postimplementation period.

The implementation of a dedicated upper extremity trauma day enabled the systematic scheduling of non-emergency surgical procedures for upper extremity fractures on a designated operating list held once-weekly. A designated group of 3 surgeons was assigned to the dedicated upper limb trauma lists, ensuring that every week one of them was available to operate from the list. The team of anesthesiologists, nurses and medical personnel remained unchanged during the entire operating day. The surgical procedures were clustered per side of injury, resulting in a series of left-sided distal radius fractures followed by a series of right-sided fractures on the same day. Positioning, draping and the surgical technique were standardized in accordance with the surgical steps described by van Veelen et al. [9]. The designated surgeons aimed to use a single implant type for all procedures during the postimplementation period whenever implant availability permitted. The aim of standardizing the workflow was to improve efficiency without compromising patient safety. All eligible consecutive patients from the postmplementation period were retrospectively assigned to the dedicated cohort for comparative analysis (Figure 2).

### 2.4. Outcomes

The primary outcomes were surgical time, operating room time, overall case time and cost of treatment. Type of procedure, surgeon and team members, the time of arrival of the patient in the anesthetic side room, arrival in the operating room, surgical procedure start and end times, the time the patient leaves the operating room, and the time of leaving the postoperative recovery room were systematically recorded by the nursing staff in the electronic health record during both anesthetic and surgical time-outs and sign-outs. Surgical time was defined as the time from incision to the last suture being placed. Operating room time was defined as the time between the patient arriving in the operating room and leaving the operating room. Overall case time was counted from the patient entering the anesthesia side room until leaving the postoperative recovery room.

The data were extracted by administrative hospital staff who were otherwise not involved in the research project. Costs were defined as direct costs associated with the operation and extracted from the hospital’s cost-accounting system for every eligible patient. This included implant cost, operating room cost (defined as the cost of operating room utilization), anesthesia cost (defined as all costs associated with performing anesthesia, including staffing costs of the anesthetist and anesthetic nurse), and staffing costs of the involved surgeons and nurses. The sum of these costs was defined as the total cost. The calculation of costs did not differ between the preimplementation and postimplementation periods. Costs were extracted in Swiss francs (CHF). No additional adjustment for inflation was made between the non-dedicated and dedicated cohorts. This methodological choice is conservative, as inflation over the full analyzed period from January 2024 to March 2025 was positive. This implies that any observed cost reduction in the dedicated cohort occurred despite rising background costs.

Secondary outcomes were time to treatment, length of hospital stay, intraoperative complications, postoperative complications, and reoperations. Time to treatment was defined as the timeframe between the date of injury and surgical treatment. If secondary dislocation was the indication for treatment, the first diagnosis of secondary dislocation was counted as time zero. Length of hospital stay was calculated based on the number of overnight stays and retrieved from the discharge summaries. Intraoperative complications were retrieved from the operation notes and discharge summaries. The occurrence of postoperative complications and reoperations was recorded at the 90-day follow-up visit.

Patients who did not attend the 90-day follow-up visit were excluded from the denominator when calculating the percentages of complications and reoperations.

### 2.5. Statistical Analysis

The statistical analysis was performed using DataTab Version 2024 (e.U., Graz, Austria) and R version 4.5.2 (R Foundation for Statistical Computing, Vienna, Austria).

A Kolmogorov–Smirnov test was used to assess the normality of variables. Variables with a normal distribution were analyzed with Student’s *t*-test and described as mean with corresponding standard deviation (SD). Skewed continuous variables were analyzed with the Mann–Whitney U test and described as medians with corresponding interquartile range (IQR) or range. Categorical variables were analyzed with Fisher’s exact test. A subgroup analysis was performed for the combined procedure of volar locked plating with ulnar styloid fixation. Differences in medians and their corresponding 95% confidence intervals (CIs) were estimated using non-parametric bootstrap resampling with 100,000 resamples. The difference was defined as the median in the dedicated cohort minus the median in the non-dedicated cohort. The alpha value for statistical significance was set to 0.05. For the complication and reoperation rates, a risk difference 95% confidence interval was calculated using the Newcombe method based on Wilson score intervals.

## 3. Results

### 3.1. Study Population and Baseline Characteristics

During the preimplementation period from January 2024 to August 2024, a total of 183 consecutive patients underwent an open reduction and internal fixation of the distal radius through a volar plate and screw osteosynthesis in a nondedicated setting. A total of 143 patients were included in the nondedicated cohort, of whom 5 patients did not attend their scheduled follow-up visit (Figure 1). During the postimplementation period from September 2024 to March 2025, a total of 116 patients underwent a distal radius volar plate and screw osteosynthesis in a dedicated setting; 104 patients were eligible for inclusion. One patient did not attend the 90-day follow-up visit (Figure 2).

The baseline characteristics of both cohorts are summarized in Table 2. There was no difference between cohorts in terms of average age and comorbidities, fracture patterns, or frequency of associated ulnar styloid fractures.

### 3.2. Primary Outcomes

Surgical time, operating room time and overall case time differed significantly between groups. The median surgical time per procedure in the dedicated cohort was 40 min (51%) shorter than in the nondedicated cohort. Median operating room time and median overall case time were reduced by 55 min (49%) and 59 min (38%), respectively (Table 3). The dispersion of surgical time in the dedicated group was reduced from a quartile deviation of 23 min in the nondedicated cohort to 7 min in the dedicated cohort (*p* < 0.001).

The median costs of treatment are shown in Table 4. After implementation of the dedicated upper extremity trauma list, costs were significantly reduced in all categories. The highest cost savings were noted in the theatre cost subcategory. The median total treatment cost per case for volar plating of an isolated distal radius fracture was reduced by 29% (*p* < 0.001).

### 3.3. Secondary Outcomes

The complication rate at the 90-day follow-up visit did not differ significantly between the nondedicated cohort (*n* = 13; 9.4%) and the dedicated cohort (*n* = 8; 7.7%) (*p* = 0.82). The risk difference (dedicated minus nondedicated cohort) was −1.7 percentage points (95% CI, −8.8 to 6.2).

The most frequent complication in the nondedicated cohort was implant malpositioning, occurring in 2.9% of cases (*n* = 4), versus none in the dedicated cohort (Table 5).

In the nondedicated cohort, six reoperations were performed, accounting for a risk of 4.3%. In the dedicated cohort, two reoperations were performed, accounting for a risk of 1.9%. This difference was not statistically significant (*p* = 0.47) (Table 6). The risk difference (dedicated minus nondedicated cohort) was −2.4 percentage points (95% CI, −7.4 to 3.0). Using an illustrative non-inferiority margin of 2 percentage points, non-inferiority could not be established for complications or reoperations because the upper confidence limit exceeded the prespecified margin.

In the nondedicated cohort, 9.8% of the patients stayed more than 2 nights in the hospital after their operation, versus 5.8% in the dedicated cohort (Table 7). The median waiting time from the date of injury until surgery significantly increased from 2 days in the nondedicated cohort to 5 days in the dedicated cohort.

### 3.4. Subgroup Analysis

Additional ulnar styloid fixation was unevenly distributed between the nondedicated and dedicated cohorts (Table 2). Additional ulnar styloid fixation increased the surgical time by 32 min in the nondedicated cohort (Table 8). In the dedicated cohort, none of the patients underwent styloid fixation. After stratification, the statistically significant difference in favor of the dedicated cohort remained unchanged for all primary outcomes (Table 8).

In the nondedicated cohort, each case was performed by a board-certified surgeon out of a group of 10 surgeons. In 30% of the procedures, a surgeon-in-training who assisted during the operation also performed key surgical steps, such as fracture reduction or plate positioning. In the dedicated cohort, each case was performed by a board-certified surgeon out of a group of three surgeons. A surgeon-in-training performed key surgical steps in 25% of the procedures in this cohort. The difference between the two groups was not statistically significant.

## 4. Discussion

### 4.1. Summary of Results

This single-institution pre- and postimplementation study demonstrated that a dedicated upper extremity trauma list is logistically feasible; reduces surgical time, operating room time, and overall case time; decreases variability in case duration; and lowers costs. No statistically significant differences in complications or reoperations were observed at 90-day follow-up. However, given the retrospective study design and limited sample size, non-inferiority of the dedicated cohort could not be established.

### 4.2. Comparison to Literature

Most authors reporting on dedicated operating rooms for orthopedic trauma studied the effects of setting up a daily unbooked operating room for a heterogeneous pool of orthopedic trauma procedures [1,10,11,12]. This reduces the frequency of after-hours surgery secondary to adding on trauma cases to elective operating lists [10].

The set-up of our study was unique, as the goal was to create homogeneous operating lists in a dedicated operating room to increase efficiency through standardization. The dedicated operating room was only used for semi-elective upper extremity trauma.

Previous reports on homogeneous dedicated trauma lists, as in our study, are scarce. Makki et al. implemented protected three-hour time slots reserved for upper extremity trauma on a twice-weekly basis [8]. Comparison of pre- and postimplementation cohorts, comprising 52 and 78 patients, respectively, demonstrated significantly decreased hospital stays and an increased patient satisfaction after implementation of the dedicated operating lists.

Chandratrey et al. studied an acute knee trauma list for locked knees secondary to meniscal tears, loose bodies, cruciate ligament ruptures, acute osteochondral fractures and acute meniscal tears [13]. In their intervention group of 53 patients, all patients were operated on by specialist knee surgeons assisted by theatre staff who were brought up to date with surgical techniques. Standardized protocols for post-operative care were introduced. They accomplished a cost reduction of 40% per case due to a decrease in duration of stay. This is similar to the total cost reduction of 29% found in our series. However, the main contributing factor to cost reduction in our series was the reduction in operating room time.

Similarly, studies on a conventional dedicated operating room for semi-elective trauma of any kind reported significant cost savings [10,14], decreased duration of stay [3,8,10], and reduced complication rates in their dedicated cohorts [11,15]. This is in accordance with the results of our series.

### 4.3. Interpretation of Results

#### 4.3.1. Time Saving

The dedicated setting for upper extremity trauma offers important advantages in terms of time management and planning.

First, surgical time and OR time were halved after implementing the dedicated upper extremity trauma list at our institution.

Second, surgical time became more consistent. The dispersion of surgical time in the dedicated group was reduced from a quartile deviation of 23 min in the nondedicated cohort to 7 min in the dedicated cohort (*p* < 0.001). This facilitates more precise planning of trauma operating lists, as reduced variability in operative duration makes operating times more predictable.

Third, the reduction in surgical time and the improvement in its consistency were achieved through the standardization of patient positioning and surgical steps. This facilitated interplay between experienced and new team members. Experienced personnel carried out procedures in a highly systematic manner. Less experienced team members could easily adopt the technical steps and seamlessly integrate into the workflow.

#### 4.3.2. Cost Reduction Factors

The greatest proportional cost savings were found in the operating room cost subcategory, resulting directly from the 49% reduction in operating room time. Nursing costs were reduced due to the shorter duration of hospital stay in the dedicated cohort.

The quartile deviation of the total cost was reduced by 1185 CHF (53%). This facilitates cost prediction and could provide health care systems with the flexibility to move from a fee-for-service model to bundled payment, capitation or a pay-for-performance model for the treatment of semi-elective surgical fractures [16].

#### 4.3.3. Duration of Stay

The average hospital stay of 2 nights results from the Swiss healthcare model. It is financially structured in a way that requires an inpatient stay to allow hospitals to cover the costs of a distal radius fracture fixation. The preplanned required stay of 2 nights might have obscured potential differences in hospital stay between the cohorts.

#### 4.3.4. Waiting Time Until Surgery

As a result of clustering upper extremity trauma on dedicated lists, the frequency of operating on these fractures was reduced from almost daily as an add-on case to once-weekly. The waiting time until surgery increased from 2 days in the nondedicated cohort (range 0–14) to 5 days in the dedicated cohort (range 0–14).

The retrospective design of our study did not allow for an assessment of the effect of treatment delay on the functional outcomes of our patients. The complication rate at final follow-up was analyzed and was in favor of the dedicated group, although statistically not significant. These results should be interpreted with caution, as the contribution of the standardized surgical set-up in the dedicated group could have eliminated a potential negative effect of treatment delay on complication risk.

Further research is required to assess whether the average treatment delay of 3 days in the dedicated cohort has an influence on the total duration of sick leave and its related societal costs.

#### 4.3.5. Institutional Strategy

The cost reduction per case has led to the decision to start scheduling the dedicated upper extremity trauma list two days per week at our institution. This is likely to reduce the median waiting time to treatment. Future research should focus on the satisfaction of the staff involved in this high-output working environment, the effects on teaching opportunities for surgeons-in-training working in this set-up, and the long-term clinical outcomes of the dedicated setting.

### 4.4. Limitations

This study has limitations related to its design. First, the observed differences in surgical time and operating room time cannot be attributed to the dedicated operating list alone. As described in the Methods section, the dedicated trauma list combined several interventions: grouping consecutive cases of volar locking plate fixation for distal radius fractures; assigning a group of three fellowship-trained surgeons to this weekly list; standardizing the workflow; and reducing the number of implants used. Similarly, conducting the dedicated trauma list at a single hospital campus, which had previously been used to treat a more homogeneous patient population, may have influenced both surgical time and operating room time. Due to the retrospective study design, it is not possible to determine which specific component of this combined set of interventions contributed most to the improvement in efficiency. Given their organizational, institutional, and practical interdependence, these interventions could not be introduced and evaluated separately. For example, assigning the same group of three surgeons to perform all distal radius fracture fixations at our institution without implementation of a weekly list, or requiring all surgeons and orthopedic scrub nurses involved in add-on cases to adopt the standardized workflow during the preimplementation period, would not have been practicable.

Second, this study concerns a pre- and postimplementation trial. There was no control group to account for extrinsic concurrent changes in hospital efficiency over the same time interval.

Third, the generalizability of our results to other institutions is limited by institutional differences, including the incidence of upper extremity fractures, referral, hospital infrastructure and resources.

Fourth, the retrospective design of our study did not allow for the collection of important additional data, including functional clinical outcomes and duration of sick leave. Future research building on this pilot study should adopt a prospective design and collect detailed information on functional outcomes and return to work. This would enable an assessment of the financial implications associated with the outpatient postoperative period, whereas our study was limited to procedure- and hospitalization-related costs only.

Fifth, although baseline characteristics showed no significant differences between the dedicated and non-dedicated cohorts, selection bias cannot be entirely excluded because propensity score matching was not performed. Of the 247 initially enrolled patients (104 in the dedicated cohort and 143 in the non-dedicated cohort), 6 patients were lost to follow-up (1 in the dedicated group [0.96%] and 5 in the non-dedicated group [3.50%]), resulting in a final analysis cohort of 241 patients. Although overall retention was high at 97.6%, potential attrition bias cannot be entirely excluded, and missing outcome data for these few patients must be acknowledged as a limitation.

## 5. Conclusions

A dedicated upper extremity trauma list, run once-weekly by a specialized team, significantly reduced surgical time, operating room time, overall case time, variability in case duration, and total cost per case for volar plate osteosynthesis of isolated closed distal radius fractures. No statistically significant differences in complications and reoperations were observed between the dedicated and nondedicated cohorts at 90-day follow-up; however, noninferiority could not be established.

## Figures and Tables

**Table 1:** Workflow during the pre- and postimplementation periods.

	Preimplementation Period	Postimplementation Period
Preoperative anesthetic assessment	Shared decision-making on type of anesthesia	
Admission	On the day of surgery in the preoperative department	
Infrastructure	Main hospital campus	Designated hospital campus
Operating list	Variety of trauma cases: both lower and upper extremity	Semi-elective surgical fractures of the upper extremity
Preparation	Side room to perform anesthesia before bringing the patient into OR; prophylactic antibiotics 30 min. before incision.	
Anesthesia	GA or LR (axillary block); consultant anesthetist or resident with close supervision.	
Installation	Patient positioning by specialized OR technician.	
Performing surgeons	10 Trauma/orthopedic surgeons, of whom 2 are fellowship-trained in upper extremity surgery.	3 Trauma/orthopedic surgeons, all fellowship-trained in upper extremity surgery.
Assistants	1 assisting orthopedic resident, 1 circulating nurse and 1 orthopedic scrub nurse.	
Used implants	Five manufacturers: Arthrex ^1^, DePuy ^2^, BoneBridge ^3^, Marquardt Medizintechnik ^4^, Medartis ^5^	Two manufacturers: Arthrex ^1^, DePuy ^2^
Postoperative management	Removable splint for a maximum of 2 weeks; ascorbic acid 1000 mg once daily for 6 weeks; hand therapy from the first week postoperatively for active mobilization.	

GA, general anesthesia; LR, locoregional anesthesia; OR, operating room. ^1^ Arthrex^®^ Titanium Volar Distal Radius Plating System (Arthrex GmbH, Munich, Germany). ^2^ VA LCP^TM^ Two-Column Volar Distal Radius Plate (DePuy Orthopaedics Inc., Warsaw, Indiana, USA). ^3^ SALGINA 2.5 mm Volar Distal Radius System (BoneBridge, Zug, Switzerland). ^4^ WINSTA-R Distal Radius System (Marquardt Medizintechnik GmbH, Spaichingen, Germany). ^5^ Distal Radius System (Medartis AG, Basel, Switzerland).

**Table 2:** Baseline characteristics of the nondedicated and dedicated cohorts. Values are expressed as the number of patients, *n* (%), unless indicated otherwise.

	Nondedicated Cohort *n* = 143	Dedicated Cohort *n* = 104	*p*-Value
Age, yr, mean ± SD (range)	60 ± 17 (18–94)	60 ± 18 (18–87)	0.89
Male	32 (21%)	23 (22%)	0.31
ASA ^1^ Class	I: 53 (37%); II: 74 (52%); III: 15 (11%); IV: 0 (0%)	I: 35 (34%); II: 57 (54%); III: 12 (12%); IV: 0 (0%)	0.86
Antiaggregant therapy	7 (5%)	11 (11%)	0.139
Anticoagulant therapy	8 (6%)	5 (5%)	0.78
Extra-articular fracture (AO 23-A)	56 (41%)	36 (35%)	0.33
Intra-articular fracture (AO 23-B/C)	87 (58%)	68 (65%)	0.79
Associated ulnar styloid fracture	75 (52%)	53 (51%)	0.60
Additional screw fixation of ulnar styloid	4 (3%)	0 (0%)	0.14
Surgery after secondary dislocation	1 (1%)	5 (5%)	0.09
Arthrex^®^	53 (37%)	99 (95%)	-
DePuy^®^ Orthopaedics	32 (22%)	5 (5%)	-
BoneBridge AG	23 (16%)	0 (0%)	-
Medartis^®^	22 (15%)	0 (0%)	-
Marquardt Medizintechnik	13 (9%)	0 (0%)	-

^1^ ASA, American Society of Anesthesiologists Physical Status Classification System (ASA) [Saklad, Doyle].

**Table 3:** Surgical, OR, and overall case time. The values reported are the median (interquartile range; range).

	Nondedicated Cohort N = 143	Dedicated Cohort N = 104	*p*-Value	Difference [95% CI]
Surgical time, min.	79 (64–110; 27–193)	39 (32–46; 23–93)	<0.01	−40 [−47, −33]
OR time, min.	113 (94–145; 55–249)	58 (52–69; 44–122)	<0.01	−55 [−62, −49]
Overall case time, min.	157 (135–184; 70–296)	98 (88–113; 56–178)	<0.01	−59 [−67, −51]

**Table 4:** Costs of treatment. The values reported are the median (interquartile range; range). Values are expressed in Swiss Francs (CHF) unless indicated otherwise.

	Nondedicated Cohort	Dedicated Cohort ^1^	Absolute Cost Savings [95% CI]	Proportional Cost Savings (%) [95% CI]	*p*-Value
Total cost	8374 (6127–10,621; range 5618–33,266)	5920 (4858–6982; range 4293–8921)	−2454 [−2810, −2100]	−29% [−34, −25]	<0.001
Implant cost	1365 (1048–1682; range 705–5532)	1264 (1077–1451; range 826–2032)	−101 [−129, −74]	−7% [−9, −5]	<0.01
Operating room cost	1355 (649–2061; range 533–3805)	810 (473–1147; range 521–1536)	−545 [−610, −485]	−40% [−45, −36]	<0.01
Anesthesia cost	1332 (803–1861; range 41–5276)	989 (680–1298; range 604–1901)	−343 [−395, −290]	−26% [−30, −22]	<0.01
Surgeon cost	1802 (1226–2378; range 894–6215)	1162 (772–1552; range 711–2631)	−640 [−725, −555]	−36% [−40, −31]	<0.01
Nursing cost	1359 (792–1926; range 585–9112)	1075 (642–1508; range 201–3079)	−284 [−350, −220]	−21% [−26, −16]	<0.01

ND, nondedicated; D, dedicated. ^1^ Cost data were unavailable for 1 patient at the time of data extraction.

**Table 5:** Number and rate of complications in both cohorts at 90-day follow-up. Values are expressed as the number of patients, *n* (%). CRPS, complex regional pain syndrome.

	Nondedicated Cohort ^1^	Dedicated Cohort ^1^	*p*-Value
Complications	13 (9.4%)	8 (7.7%)	0.82
Loss of reduction	3 (2.2%)	3 (2.9%)	1.00
Implant malpositioning	4 (2.9%) ^2^	0	0.14
Nerve injury to sensory branch of radial nerve	1 (0.7%)	2 (1.9%)	0.58
Carpal tunnel syndrome	2 (1.4%)	1 (1.0%)	1.00
CRPS	3 (2.2%)	1 (1.0%)	0.64
Peri-implant fracture	1 (0.7%)	0	1.00
Ulnar impaction syndrome	0	1 (1.0%)	0.43
Infection	0	0	-

^1^ Patients who did not attend the 90-day follow-up visit were excluded from the denominator when calculating the percentages of complications and reoperations. ^2^ Intra-articular positioning of one or more screws through the volar locking plate (*n* = 2); pain due to the ulnar styloid screw (*n* = 2).

**Table 6:** Number and rate of reoperations in both cohorts at 90-day follow-up. Values are expressed as the number of patients, *n* (%).

	Nondedicated Cohort	Dedicated Cohort	*p*-Value
Reoperation overall	6 (4.3%)	2 (1.9%)	0.47
Revision for loss of reduction	1 (0.7%)	1 (1.0%)	1.00
Removal of metalwork for malpositioning	3 (2.2%)	0	0.26
Removal of metalwork for carpal tunnel syndrome	1 (0.7%)	0	1.00
Isolated carpal tunnel release (without removal of metalwork)	0	1 (1.0%)	0.43
Revision for peri-implant fracture	1 (0.7%)	0	1.00

**Table 7:** Hospital stay and time until treatment.

	Nondedicated Cohort N = 143	Dedicated Cohort N = 104	*p*-Value
Median length of stay, days	2 (2–2; 1–15)	2 (2–2; 1–3)	-
Hospital stay > 2 nights, *n* (%)	*n* = 14 (9.8%)	*n* = 6 (5.8%)	0.35 ^1^
Time until surgery, days	2 (1–5; 0–14)	5 (4–7; 0–14)	<0.01 ^2^

^1^ Fisher’s exact test. ^2^ Mann–Whitney U test.

**Table 8:** Subgroup analysis. Median surgical time, OR time, overall case time, and implant cost in patients with and without ulnar styloid fixation. The p-value was calculated for comparison of the groups ND, without ulnar styloid fixation, and D, without ulnar styloid fixation.

		Surgical Time	OR Time	Overall Case Time	Implant Cost
ND overall	*n* = 143	85 (range 27–193)	120 (range 55–249)	162 (range 70–296)	1410 (range 705–5532)
ND without ulnar styloid fixation	*n* = 139	79 (range 27–193)	113 (range 55–249)	157 (range 55–249)	1406 (range 705–5532)
ND with ulnar styloid fixation only	*n* = 4	111 (range 79–130)	154 (range 138–163)	209 (range 154–251)	1531 (range 1212–1826)
D without ulnar styloid fixation	*n* = 104	39 (range 23–93)	58 (range 44–122)	98 (range 56–178)	1269 (range 826–2032)
*p*-value		<0.01	<0.01	<0.01	<0.01

## Data Availability

The original contributions presented in this study are included in the article. Further inquiries can be directed to the corresponding author.

## References

[1] Bhattacharyya T., Vrahas M.S., Morrison S.M., Kim E., Wiklund R.A., Smith R.M., Rubash H.E. (2006). The value of the dedicated orthopedic trauma operating room. J. Trauma.

[2] World Health Organization (2001). The International Classification of Functioning Disability Health: ICF.

[3] Heng M., Wright J.G. (2013). Dedicated operating room for emergency surgery improves access and efficiency. Can. J. Surg..

[4] Meneghini R.M. (2021). Techniques and Strategies to Optimize Efficiencies in the Office and Operating Room: Getting Through the Patient Backlog and Preserving Hospital Resources. J. Arthroplast..

[5] Sershon R.A., Ast M.P., DeCook C.A., Della Valle C.J., Hamilton W.G. (2024). Advanced Concepts in Outpatient Joint Arthroplasty. J. Arthroplast..

[6] Stepaniak P.S., Vrijland W.W., de Quelerij M., de Vries G., Heij C. (2010). Working with a Fixed Operating Room Team on Consecutive Similar Cases and the Effect on Case Duration and Turnover Time. Arch. Surg..

[7] Lo E.Y., Bowler J., Lines T., Melton C., Volkmer R., Majekodunmi T., Krishnan S.G. (2021). Operating room efficiency and cost reduction in shoulder arthroplasty: Is there an advantage of a dedicated operating room team?. Semin. Arthroplast. JSES.

[8] Makki D., Alnajjar H.M., Saw N. (2016). Development and efficiency of dedicated upper limb trauma lists in a district general hospital. J. Perioper. Pract..

[9] van Veelen N.M., Babst R., Link B.C., van de Wall B.J.M., Beeres F.J.P. (2023). Distale Radiusfrakturen–Taktik und Zugangswahl [Distal radius fracture-tactic and approach]. Oper. Orthop. Traumatol..

[10] Brusalis C.M., Shah A.S., Luan X., Lutts M.K., Sankar W.N. (2017). A Dedicated Orthopaedic Trauma Operating Room Improves Efficiency at a Pediatric Center. J. Bone Jt. Surg. Am..

[11] Lemos D., Nilssen E., Khatiwada B., Elder G.M., Reindl R., Berry G.K., Harvey E.J. (2009). Dedicated orthopedic trauma theatres: Effect on morbidity and mortality in a single trauma centre. Can. J. Surg..

[12] Wixted J.J., Reed M., Eskander M.S., Millar B., Anderson R.C., Bagchi K., Kaur S., Franklin P., Leclair W. (2008). The effect of an orthopedic trauma room on after-hours surgery at a level one trauma center. J. Orthop. Trauma.

[13] Chandratreya A.P., Spalding T.J., Correa R. (2006). Development and efficiency of an acute knee trauma list. Injury.

[14] Cloud C., Fong B., Bloise C., Leonardi C., Krause P.C., Lee O.C. (2022). Advantages of a Dedicated Orthopaedic Trauma Operating Room for Diaphyseal Femur Fractures. J. Orthop. Trauma.

[15] Roberts T.T., Vanushkina M., Khasnavis S., Snyder J., Papaliodis D.N., Rosenbaum A.J., Uhl R.L., Roberts J.T., Bagchi K. (2015). Dedicated orthopaedic operating rooms: Beneficial to patients and providers alike. J. Orthop. Trauma.

[16] Gosden T., Forland F., Kristiansen I.S., Sutton M., Leese B., Giuffrida A., Sergison M., Pedersen L. (2000). Capitation, salary, fee-for-service and mixed systems of payment: Effects on the behaviour of primary care physicians. Cochrane Database Syst. Rev..

